# The immunoproteasome in Alzheimer’s disease: a dual regulator of tau pathology and microglial senescence

**DOI:** 10.1007/s10787-026-02393-1

**Published:** 2026-10-03

**Authors:** Mustafa M. Shokr, Ahmed M. Abdelaziz

**Affiliations:** Department of Pharmacology and Toxicology, Faculty of Pharmacy, Sinai University – Arish Branch, Arish, 45511 Egypt

**Keywords:** Immunoproteasome, LMP7, Microglial senescence, Tauopathy, Neuroinflammation, Alzheimer's disease

## Abstract

Alzheimer’s disease (AD) is characterized by progressive cognitive decline driven by the convergence of tauopathy, impaired proteostasis, and chronic neuroinflammation. Emerging evidence identifies the immunoproteasome (iP), particularly the β5i/LMP7 subunit, as a central regulator linking these pathological processes. Unlike the constitutive proteasome, the iP is markedly induced in microglia, neurons, and astrocytes under inflammatory conditions through interferon-γ and NF-κB signaling. This review discusses the dual and context-dependent role of the iP in AD pathogenesis. In microglia, chronic iP activation promotes degradation of NRF2, leading to oxidative stress, NLRP3 inflammasome activation, and the development of a senescence-associated secretory phenotype that exacerbates neuroinflammation and tau propagation. In neurons, moderate iP activity facilitates the clearance of phosphorylated tau; however, sustained overactivation generates aggregation-prone tau fragments that enhance trans-synaptic tau spreading. We further highlight the cross-talk between the iP and autophagy pathways through TFEB and p62/SQSTM1 signaling and discuss how gut microbiota-derived inflammatory mediators may prime central iP activation through the gut–brain axis. Importantly, recent preclinical studies support the concept of partial immunoproteasome modulation rather than complete inhibition, with selective LMP7 targeting restoring proteostatic balance, reducing microglial senescence, and attenuating tau pathology. Collectively, the iP emerges as a promising immunopharmacological target and a potential therapeutic rheostat in AD.

## Introduction

Conceptualized under the amyloid cascade hypothesis for more than two decades, the pathology of AD remains unsatisfactory following the development of only minor improvements through clinical trials aimed at targeting Aβ. Therefore, efforts must now be made to tackle all aspects of the triad of AD, such as failed clearance of protein aggregates, trans-synaptic propagation of tau pathology, and chronic inflammation (Alshammari et al. 2025; Alameen et al. 2026). The accumulation of Aβ aggregates outside neurons induces cellular stress response, thus exceeding microglial phagocytic potential, while p-tau aggregates form inside neurons and propagate themselves in a hierarchical manner from the entorhinal cortex to the hippocampus and neocortex (Alshammari et al. 2025; Alqahtani et al. 2025). Interestingly, cognitive decline is associated with tauopathy rather than with Aβ pathology. What is more important is that the two pathogenic aspects are not independent but, instead, dependent on each other since Aβ increases tau pathology through microglia-dependent mechanisms, and the latter increases vulnerability to neuronal inflammation (Alshahrani et al. 2025; Shokr 2025a). Chronic neuroinflammation, which occurs mainly due to senescent/dystrophic microglia, creates a vicious cycle in which pro-inflammatory cytokines (IL-1β, IL-6, and tumor necrosis factor (TNF) and complement components contribute further to the inhibition of Aβ removal and tau hyperphosphorylation through p38 MAP kinase and cyclin-dependent kinases 5 (Alshahrani et al. 2025). Together, failure of proteostasis, prion-like tau propagation, and chronic unresolved inflammation constitute the pathophysiological triad at the heart of AD (Bhatia and Godad 2025).

### The proteasome system in AD: why the constitutive proteasome is downregulated while iP is induced

**Fig. 1:**
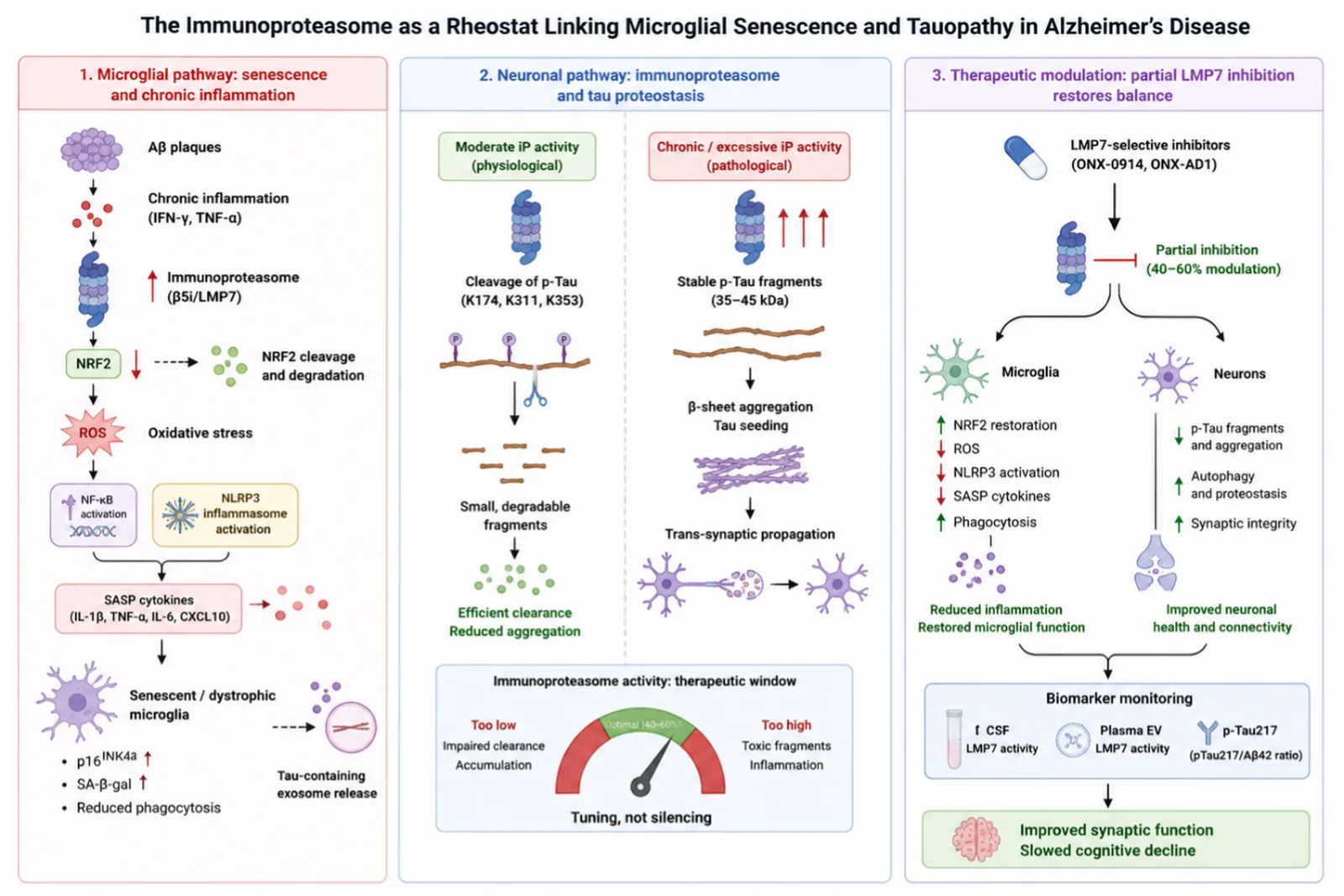
The immunoproteasome acts as a central rheostat linking neuroinflammation, microglial senescence, and tau pathology in AD. In microglia, chronic exposure to Aβ and inflammatory cytokines induces β5i/LMP7 immunoproteasome activity, resulting in NRF2 degradation, oxidative stress, NLRP3 activation, and senescence-associated secretory phenotype (SASP) formation. Senescent microglia exhibit impaired phagocytosis and release tau-containing exosomes that promote tau propagation. In neurons, moderate immunoproteasome activity facilitates clearance of phosphorylated tau, whereas chronic overactivation generates aggregation-prone tau fragments and trans-synaptic spreading. Partial LMP7 inhibition (40–60%) restores proteostatic balance, reduces inflammation and tau aggregation, and improves neuronal integrity, supporting the concept of “tuning, not silencing” immunoproteasome activity in AD

The ubiquitin-proteasome pathway is the key protease responsible for the degradation of short-lived, abnormal, or damaged intracellular proteins (Bhat et al. 2022). While constitutive proteasomes (cP) regulate proteostasis via degradation of ubiquitin-tagged substrates, such as normal tau, the expression of the cP in AD brain specimens was observed to be significantly reduced due to chymotrypsin-like and trypsin-like enzymatic activities, along with the presence of polyubiquitinated protein accumulations in neurofibrillary tangles and dystrophic neurites (Bhat et al. 2022) **[**Figure 1**].** This counterintuitive phenomenon of reduced cP expression, despite the presence of greater proteotoxic load, may occur due to the oxidation of the proteasomal subunits, inhibition by Aβ oligomers, and a decrease in proteasomal assembly factors due to aging (Ruiz-Romero et al. 2024). Thus, the decreased capacity for proteasomal function allows for the accumulation of toxic proteins, thereby increasing the proteotoxic load in the cells. In marked contrast, the immunoproteasome (iP), a variant of proteasomes, which consists of inducible subunits of β1i/LMP2, β2i/MECL-1, and β5i/LMP7, demonstrates an enhanced expression in AD brain specimens (Ruiz-Romero et al. 2024).

The mechanism is fueled by chronic inflammation, mediated by the cytokines interferon gamma (IFN-γ) and tumor necrosis factor alpha (TNF-α), and triggers transcription factors such as STAT1 and nuclear factor kappa B (NF-κB), resulting in the production of the iP subunit genes, PSMB8, PSMB9, and PSMB10 (Alanazi et al. 2023). The iP, unlike the cP, maximizes the production of MHC Class I peptides and is highly efficient in cleaving hydrophobic and basic amino acids; however, the iP lacks efficacy in processing oxidized and aggregated proteins (Rock et al. 2014). Hence, the AD brain suffers from the maladaptive proteasome shift, characterized by reduced expression of the cP, responsible for the breakdown of tau and Aβ precursors, and increased expression of the inappropriate iP, which continues the inflammatory process (Bonet-Costa et al. 2016).

### iP is a druggable node at the intersection of inflammation and tauopathy

The concurrent induction of the iP in AD and its unique localization at the crossroad of neuroinflammation and tau proteostasis render it a uniquely appealing target from the standpoint of pharmacotherapy (Zhao et al. 2026) **[**Figure 1**].** Unlike the constitutive proteasome, which cannot be fully inhibited due to the fact that it is essential for basic cellular survival, the iP can be spared by the organism since mice deficient for any of its subunits survive normally (Zhao et al. 2026). Notably, in addition to its role in innate immunity, recent findings uncovered another aspect of iP function in AD, which is contingent on cell type and proteinase activity level (Zhao et al. 2026). In microglia, iP activity leads to proteolysis of NRF2, a critical transcription factor of antioxidant response, thus contributing to oxidative stress and senescence-associated secretory phenotype (SASP) of microglia, which causes bystander neuronal dysfunction (Velagapudi et al. 2018; Shokr 2025b).

Treatment with the iP subunit inhibitor (β5i/LMP7) resulted in increased NRF2 levels, reduced activation of NLRP3 inflammasome, and microglial senescence reversal without their elimination (Velagapudi et al. 2018). On the other hand, in neurons, iP activity acts in an opposing manner; namely, at normal levels, iP preferentially cleaves hyperphosphorylated tau at specific lysine residues (Perluigi et al. 2024). Nevertheless, under chronic overstimulation by inflammation cues, the iP produces stable, self-aggregable tau fragments that trigger pathology and spread to nearby cells (Perluigi et al. 2024). This dual role implies that the strategy for AD therapy should neither suppress nor enhance iP function entirely but rather tune iP activity. This means decreasing iP activity in microglia to prevent cellular senescence and increasing iP activity in neurons to ensure tau removal (Perluigi et al. 2024). The idea of tuning iP activity is now feasible using drugs that can inhibit specific subunits of the iP, especially LMP7 subunit selective inhibitors, in the central nervous system (Chen et al. 2021). As such, the iP is the first-of-a-kind pathway wherein modulation of enzymatic activity has the potential to mitigate neuroinflammation, improve proteostasis, and block tau propagation (Chen et al. 2021).

## Cell-type specific expression and induction of iP in AD brain

### Microglia

Single-nucleus and single-cell RNA sequencing have provided unprecedented insights into microglial heterogeneity in AD, demonstrating that microglia are not activated uniformly but exhibit different transcriptional profiles in response to local pathology (Garg et al. 2025). Among the most convincing observations are those concerning the induction of PSMB8 (β5i/LMP7), the iP subunit gene, in two types of microglia: DAM and IRM. DAMs were originally described in mice with amyloidosis and defined by decreased homeostatic marker expression (P2RY12, TMEM119, CX3CR1) and increased phagocytosis and lipid metabolism (APOE, TREM2, LPL, CST7) (Garg et al. 2025). In this particular DAM population, PSMB8 was one of the most highly upregulated transcripts, with up to a 10- to 20-fold increase compared to homeostatic microglia (Guo et al. 2022). These results were corroborated in AD patients’ postmortem brain samples, where PSMB8-positive microglia accumulated near amyloid plaques and neurons with intracellular tau deposition (Guo et al. 2022).

Secondly, the population IRM is characterized by a type I interferon signature (IFIT1, IFIT2, IFIT3, MX1, ISG15), which is highly represented in regions with an ongoing spreading of tau proteins (Sanford and McEwan 2022). In IRM, the expression of PSMB8 is mediated by IFN-γ signaling from the infiltrating T cells and neuronal release of nucleic acid fragments associated with tau that activate TLR7/8 receptors (Sakatani et al. 2022). It should be noted that the DAM and IRM phenotypes are not exclusive; the DAM-IRM population is associated with the highest expression of PSMB8 and demonstrates the strongest SASP features (Sakatani et al. 2022). Regarding the functional role of PSMB8 in microglia, there is evidence of a positive feedback loop: iP-dependent proteolysis of the inhibitor NRF2 leads to activation of NF-κB, promoting further transcription of PSMB8 and pro-inflammatory factors (Gado et al. 2023). This cycle results in the sustained level of microglial iP activity even after elimination of external stimulus, which contributes to chronic and non-resolving neuroinflammation observed in AD (Hennig et al. 2018). The level of PSMB8 expression in microglia correlated with the accumulation of amyloid plaques and cognitive impairment in different Braak stages, and thus, microglial iP may be used as a marker of disease progression (Long et al. 2022).

From a therapeutic standpoint, this highly specific, selective upregulation of PSMB8 within both DAM and IRM microglia may actually be advantageous because the potential pharmacological inhibition of the iP would be specifically directed toward these particular pro-inflammatory microglia, whereas other microglia with constitutive proteasome activity would be unaffected (Guo et al. 2022). In addition, microglial cells do not express PSMB8 at equal levels because there is higher expression of PSMB8 by microglia associated with plaques than by those not associated with plaques, and females have higher expression of PSMB8 than male individuals with AD. The iP is therefore critical for microglial state changes in AD (Guo et al. 2022).

### Neurons

Neurons have unique characteristics compared to other CNS cells in terms of their low constitutive expression levels of the iP components. Basically, neurons utilize the constitutive proteasome solely for protein degradation. PSMB8, PSMB9, and PSMB10 transcript levels in neurons are barely detected by in situ hybridization or single-nucleus RNA sequencing (Woo et al. 2025). Such a phenomenon may be attributed to evolutionary selection pressure. Neurons are long-lived, post-mitotic cells that need to prevent unwanted inflammation and conserve protein degradation capacity for indispensable maintenance processes (Woo et al. 2025).

Nonetheless, this situation drastically changes in the AD brain. Single-nucleus RNA-seq analysis shows that PSMB8 expression levels increase in a particular class of neurons with neurofibrillary tangles in the AD human hippocampus (Oxendine et al. 2025). In such a way, neurons use two factors to trigger iP formation. First, p-Tau accumulation initiates neuron iP activation. At the molecular level, p-Tau aggregates recruit ATF6, a component of the unfolded protein response (UPR) pathway, thus inducing transcription of PSMB8 in the presence of the ER stress response element (Song et al. 2024). This results in a negative feedback loop, whereby a certain level of iP induction aids in the degradation and clearance of p-Tau fragments, while over-induction produces toxic tau fragments (Song et al. 2024). The second factor contributing to iP induction in neurons is endoplasmic reticulum (ER) stress. It is a common feature of vulnerable neurons in AD patients, and several factors, including impaired calcium homeostasis, oxidative stress, and the buildup of unfolded proteins, cause ER stress, resulting in activation of the three UPR receptors IRE1α, PERK, and ATF6, all capable of inducing PSMB8 (Ghemrawi and Khair 2020).

Of significance is the fact that p-Tau also contributes to the development of ER stress by disrupting ER to Golgi transport, thereby forming a positive feedback mechanism in which p-Tau and ER stress work together to induce iP expression (Song et al. 2024). Additionally, single-cell analysis indicates that iP expression does not occur homogeneously among neurons, and excitatory neurons in the entorhinal cortex and CA1 hippocampus region show higher levels of iP induction compared to other neurons, a pattern associated with their increased susceptibility to p-Tau aggregation (Weidling et al. 2025). Inhibitory interneurons have little to no increase in iP induction, suggesting why they are less prone to tau pathology (Weidling et al. 2025). Nonetheless, under conditions of severe neuronal disease and continuous ER stress, the iP is maladaptive, leading to stable fragments of tau proteins that cannot be degraded and spread trans-synaptically (Weidling et al. 2025).

### Astrocytes

Astrocytes were long thought to be simple bystanders supporting neuronal homeostasis; however, recent studies demonstrate their importance as active contributors to neuroinflammation and antigen presentation during AD progression (Zhao et al. 2024; Rajab et al. 2025). The analysis of the iP subunit expression in AD patients revealed that astrocytes exhibit a unique pattern of PSMB8, PSMB9, and PSMB10 protein abundance that is not shared by healthy neurons and reactive microglia (Zhao et al. 2024). Thus, the intermediate profile of expression of the studied proteins implies the secondary participation of astrocytes in iP-dependent degradation of target proteins during AD (Zhao et al. 2024). At the same time, there are several reasons to believe that the astrocyte contribution to AD development is independent of that of microglia (Zhao et al. 2024). Single-nucleus RNA-seq showed that some astrocytes enhance the expression of iP subunits depending on the presence of both amyloid plaques and tau lesions (Smit et al. 2021).

The second, and arguably the most significant, role of iP expression in astrocytes is its role in antigen presentation. Whereas in microglia, increased levels of iP are involved in the regulation of NLRP3 activity and oxidative responses, in astrocytes, iP is used for the synthesis of high-affinity peptides for association with MHC Class I molecules (HLA-A, B, C) (Grotemeyer et al. 2022). Reactive astrocytes near plaques in post-mortem AD tissue demonstrate the presence of PSMB8 and MHC Class I on the surface of their cells, something which is very rare for non-pathological brain (Price et al. 2021). In addition, astrocytes may present antigens relevant to AD, such as tau protein-derived peptides and fragments of apolipoprotein E, to CD8 + T cells invading the AD brain (Price et al. 2021).

It is well documented that recently discovered clonally expanded CD8 + T cells in AD patients’ CSF and parenchyma respond to epitopes presented by astrocytes (Ohyagi et al. 2025). Astrocytic antigen presentation could theoretically assist in clearing dysfunctional or infected neurons, but could also initiate T-cell-mediated cytolysis of healthy neurons and astrocytes, a mechanism involved in other forms of neurodegeneration (Li et al. 2019). Notably, single-nucleus analyses indicate that the astrocytic antigen presentation pathway is activated in higher levels of Braak staging (V-VI), paralleling significant T-cell invasion (Li et al. 2019). Therapeutically, targeting astrocytic iP poses novel considerations (Czyżewski et al. 2024). Since astrocytic iP contributes minimally to total CNS proteostatic capacity compared to microglial and neuronal iP, inhibition could be more feasible; however, complete abrogation of astrocytic iP could interfere with beneficial immune monitoring in the aged brain (Czyżewski et al. 2024). Currently, there are no astrocyte-targeting genetic methods for experimentation, but innovative adeno-associated virus-based approaches aimed at astrocyte-specific promoters (GFAP, ALDH1L1) can provide solutions shortly (Gleichman et al. 2023). As of yet, there is general agreement that astrocytic iP has a minor but important impact on AD pathogenesis, mediating immune interactions by antigen presentation through MHC-I molecules (Stanca et al. 2023). As such, any therapeutic strategy attempting to modulate the iP in AD must consider not only microglial and neuronal effects but also the potential immunological consequences of altering astrocytic antigen presentation (Stanca et al. 2023).

## Novel pathway 1: iP-driven microglial senescence and SASP

The groundbreaking finding in the field of AD involves the identification of chronic iP activation as the cause of microglial senescence, a phenomenon characterized by permanent cessation of the cell cycle associated with a harmful SASP, via an entirely novel pathway, which revolves around the transcription factor NRF2 (Chu et al. 2024). Under normal circumstances, NRF2 functions as the main coordinator of the body’s antioxidant response by entering the nucleus in response to oxidative stress to induce numerous cytoprotective enzymes such as heme oxygenase-1 (HO-1), NAD(P)H quinone dehydrogenase 1 (NQO1), and glutathione S-transferases (Chu et al. 2024). Nonetheless, in the diseased brain, constant stimulation by amyloid-beta (Aβ), tau oligomers, and pro-inflammatory factors leads to continuous expression of the iP, which has been proven to be capable of cleaving and eliminating NRF2 in an ubiquitination-independent manner, particularly through the β5i/LMP7 subunit (Zhao et al. 2026). Unlike the constitutive proteasome, which requires prior ubiquitination via the Keap1 pathway for NRF2 degradation, the iP targets a unique PEST-like sequence within the Neh6 region of NRF2, specifically cleaving at tyrosine and arginine sites (Baird and Yamamoto 2020). The result of such degradation of NRF2 due to iP is severe disruption of the antioxidant system of the cells. Reactive oxygen species (ROS) begin to build up inside microglia, and the oxidative stress results in lipid peroxidation, protein carbonylation, and DNA damage (Ashok et al. 2022). An oxidative state inside cells becomes an important stimulus for activation of NF-κB, the key transcription factor responsible for initiation of transcription of genes encoding inflammatory mediators (Lingappan 2018). Because NRF2 cannot compete with NF-κB for binding to CREB-binding protein/p300, the latter remains unregulated and begins to induce the synthesis of IL-1β, TNF-α, and CXCL10 -the main components of the microglia SASP (Wardyn et al. 2015). Inflammatory signaling mediated by these three molecules creates paracrine signaling that affects both neurons and astrocytes and recruits peripheral macrophages to enter the brain (Kölliker-Frers et al. 2021). However, aside from the induction of inflammation, the consequences of microglia aging include important changes in the phenotype of senescent microglia. Such cells can be characterized by expression of cell cycle inhibition markers like p16^INK4a and p21^CIP1/WAF1 (Carr et al. 2025). They reduce the expression of phagocytic markers such as TREM2, CD36, and CX3CR1, thus becoming unable to phagocytose and break down the fibrillar Aβ aggregates present in the vicinity of senile plaques (Carr et al. 2025).

Additionally, senescent microglia function as factories that produce exosome-based propagation of tau protein. By mechanism, senescent microglia release exosomes containing hyperphosphorylated forms of tau proteins along with microRNAs and proteases into the extracellular milieu, where they are absorbed by adjacent neurons (Sharma et al. 2026). The exosomal tau becomes a seed for misfolding and trans-synaptic propagation of native tau (Sharma et al. 2026). Causal evidence supporting the involvement of iP activity in the triggering of the senescence program has been clearly provided by employing the selective LMP7 inhibitor ONX 0914 (von Brzezinski et al. 2017). Treatment of the 5xFAD mouse, which expresses five familial mutations leading to AD and is characterized by a pronounced accumulation of Aβ, with a CNS-penetrant concentration of ONX 0914 for two months, led to an impressive decrease by 60–70% in the number of senescent microglia expressing p16^INK4a, normalization of SA-β-gal activity, and recovery of homeostatic microglial markers (Dinkins et al. 2015). It is noteworthy that this therapy was accompanied by lower exosome-associated levels of tau protein in the interstitial fluid and decreased spreading of tau protein from the injected hippocampus to the contralateral hemisphere (Lopes et al. 2022). All these data support the existence of a cascade starting from chronic activation of the iP, leading to the breakdown of NRF2, oxidative stress, NF-κB activation, induction of microglial senescence, impairment of Aβ phagocytosis, and release of tau protein-containing exosomes. The iP plays the role of the key regulator of the senescent microglia, providing an excellent mechanistic justification for the usage of partial LMP7 inhibition as a senomorphic strategy in AD (Chen et al. 2021).

## Novel pathway 2: the tau proteostasis paradox

It is worth mentioning that there is a unique and unexpected dual nature of the activity of the iP in terms of the tau proteostasis (Zhao et al. 2026). On one hand, the iP at moderate activity removes hyperphosphorylated tau from neurons, thus protecting them from neurodegenerative processes (Zhao et al. 2026). But on the other hand, under high and/or prolonged activity, the same iP becomes neurotoxic, creating seeds for further aggregation of tau fragments. This phenomenon is called the paradox of tau proteostasis because of the differences between the cleavage specificity of the iP and cP (Zhao et al. 2026). While the cP prefers to cleave the substrates after hydrophobic and basic residues, producing peptide products which can be quickly transformed into amino acids, the iP demonstrates increased cleavage specificity, being able to cleave after the hydrophobic and branched chain residues as well as after specific lysines (K) in the protein substrates (Zhao et al. 2026).

In cases where there is moderate induction of iP, like those happening when there is stress response to soluble p-Tau oligomers, the iP specifically cleaves hyperphosphorylated tau at lysine sites that include K174 (in the proline-rich domain), K311 (in the microtubule binding repeat domain), and K353 (Xia et al. 2023). Such cleavage leads to the production of small-sized fragments of tau proteins measuring between 15 and 25 kDa, which are degraded by peptidases without any aggregation ability (Xia et al. 2023). Besides, by destroying p-Tau that may aggregate to form oligomers, the process prevents further aggregation that leads to seed generation for more normal tau molecules to change into pathological forms (Xia et al. 2023). This way, iP acts as quality control of tau proteins, complementing constitutive proteasome activity, which gradually gets disrupted in aged AD brains due to oxidative insults and inhibition by Aβ oligomers. However, under chronic overactivation of the iP, cleavage of p-Tau becomes pathological (Maphis et al. 2015). In conditions where inflammation is sustained for weeks to months due to IFN-γ from T cells and TNF-α from microglia, chronic high iP activation takes place in neurons (Maphis et al. 2015). In this case, iP does not end up generating small fragments. Rather, the higher density of activated iP results in a processive activity that results in protease-resistant tau fragments with defined molecular weight, which includes a 35-kDa fragment that contains tau residues 151–391 and a 45-kDa N-terminal fragment missing its C-terminal part (Lyu et al. 2021). Interestingly, these fragments is not only their resistance to further degradation by proteasomes due to a lack of exposed lysine residues needed for ubiquitination, but also the tendency of their beta-sheet structures to aggregate, resembling paired helical filaments present in AD patient brains (Lyu et al. 2021).

Importantly, the tau proteostasis paradox translates directly into a therapeutic hypothesis: instead of targeting the iP enzyme itself, the aim would be to regulate the activity of the iP within a therapeutic range, allowing the degradation of neuron-derived p-Tau without creating stable seed-competent species (Shin et al. 2021). This idea of partial iP inhibition has recently been corroborated experimentally in the Tau-P301S mouse model using ONX 0914, wherein a low dose resulted in a roughly 50% reduction of insoluble p-Tau levels, as well as a lack of seeding activity in biosensor assays, while a higher dose and full inhibition paradoxically induced aggregation (Zhou et al. 2021). Overall, the comprehension and utilization of the tau proteostasis paradox represents a breakthrough within the scope of immunopharmacology for AD.

## Cross-talk between iP and autophagy pathways

**Fig. 2:**
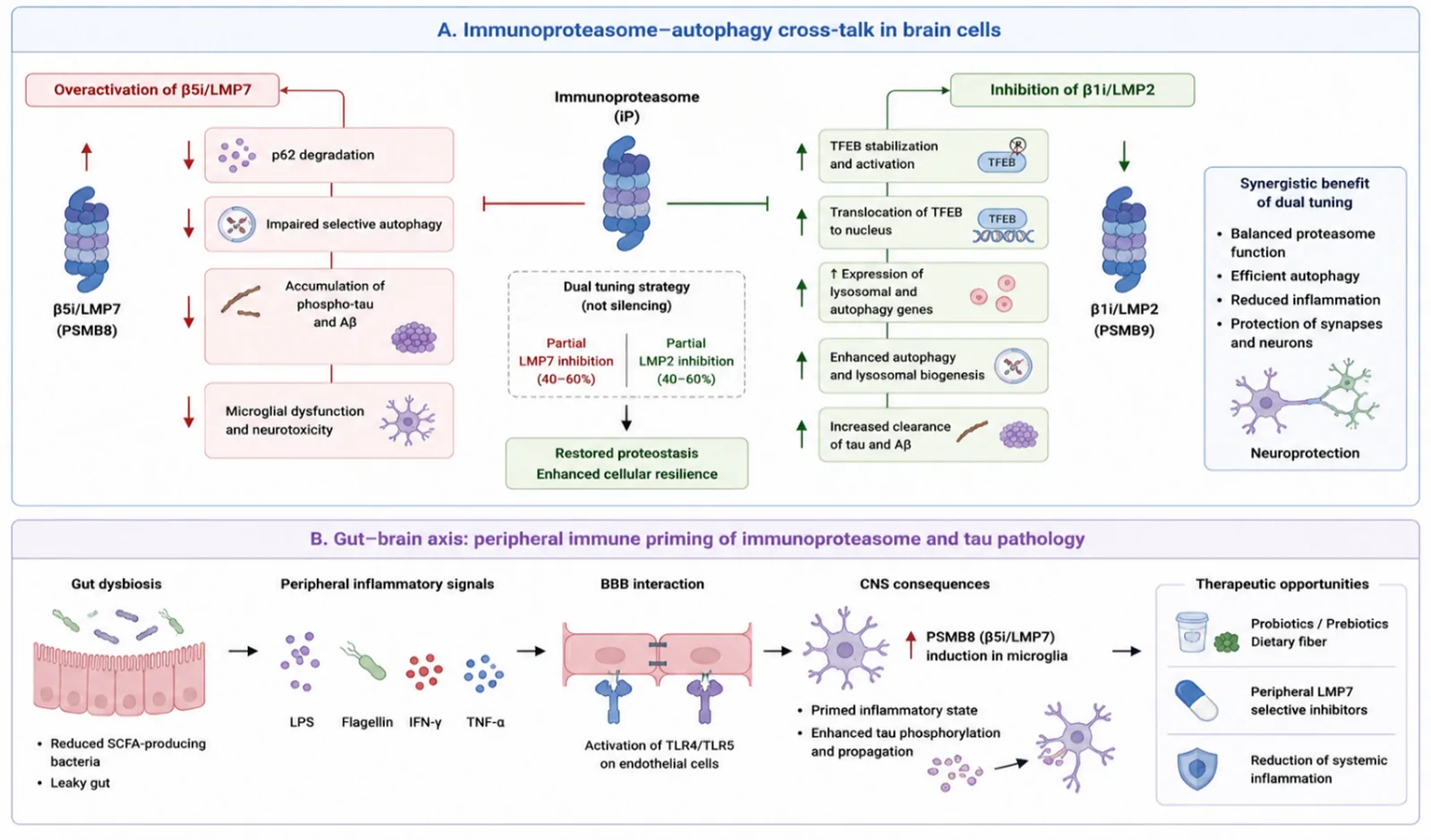
Cross-talk between the immunoproteasome, autophagy pathways, and the gut–brain axis in Alzheimer’s disease. Overactivation of β5i/LMP7 promotes degradation of p62/SQSTM1, resulting in impaired selective autophagy, accumulation of phosphorylated tau and Aβ aggregates, and microglial dysfunction. In contrast, partial inhibition of β1i/LMP2 stabilizes TFEB, enhances its nuclear translocation, and induces lysosomal and autophagy-related genes, thereby improving clearance of tau and amyloid aggregates. Combined partial inhibition of LMP7 and LMP2 restores proteostatic balance and promotes neuroprotection. The lower panel illustrates how gut dysbiosis and reduced short-chain fatty acid production increase systemic inflammatory mediators, including LPS, IFN-γ, and TNF-α, which activate endothelial TLR signaling and prime microglial immunoproteasome expression, thereby accelerating tau propagation and neuroinflammation in AD

Although, the iP does not function in a standalone mode, it still interacts with another important intracellular clearance mechanism called the autophagy-lysosome system, forming the proteostatic axis that is deregulated in AD (Bustamante et al. 2018) **[**Figure 2**].** Therefore, deciphering the nature of cross-talk between these two mechanisms is vital, as any drug therapy for modulating iP can potentially affect autophagy or vice versa. This cross-talk depends primarily on the action of TFEB, a transcription factor that is the key regulator of lysosomal biogenesis and autophagy. In the basal state, TFEB remains phosphorylated via mTORC1 signaling and is sequestered in the cytoplasm; however, during nutritional deprivation and lysosomal stress, it gets dephosphorylated, migrates into the nucleus, and activates the expression of the genes responsible for the formation of autophagosomes (LC3, ATG5, ATG7) and lysosomes (cathepsin, glucocerebrosidase, LAMP1, LAMP2) (Bustamante et al. 2018). It has been recently shown that TFEB itself is degraded via the ubiquitin-proteasome system and specifically by the iP. In particular, the β1i/LMP2 subunit has been shown to play a role in TFEB degradation (Kim et al. 2026): TFEB protein levels are elevated by a factor of three in microglia from knockout mice deficient in LMP2, which results in increased autophagic flux and more rapid clearance of Aβ and tau aggregates (Kim et al. 2026). This phenomenon has been capitalized upon pharmacologically with the use of the specific LMP2 inhibitor LU-002i, which elevates nuclear TFEB, promotes LC3-II lipidation, and reduces p-Tau aggregates in Tau-P301S mice by around 30%, an effect additive with LMP7 inhibition (Wang et al. 2016). On the other hand, prolonged β5i/LMP7 activation in AD microglia leads to degradation of the autophagy receptor p62/SQSTM1, which facilitates linking of ubiquitinated cargo with LC3 in autophagosomes (Zhou et al. 2024). The degradation of p62 compromises selective autophagy of aggregated proteins such as tau oligomers, thus perpetuating the vicious circle wherein the malfunction of the proteasome cannot be complemented by autophagic elevation (Ono et al. 2022).

Apart from TFEB and p62, other points of crosstalk between the iP and autophagy involve the deubiquitinating enzyme USP14, which controls both proteasomal and autophagic pathways, and the ER stress receptor IRE1α, which activates iP subunits and autophagy factors through the XBP1 splice variant (Ji and Kwon 2017). In terms of clinical application, this knowledge allows for the concept of dual tuning, namely, selective inhibition of LMP7 (at 40–60%) to decrease NRF2 turnover and microglia senescence, coupled with LMP2 (30–50%) to boost TFEB-induced autophagy (Brackhan et al. 2023).

## The gut-brain axis: peripheral priming of CNS iP

The recently recognized gut-brain axis opens a novel, yet overlooked, aspect of iP biology in AD (Hasan et al. 2025) **[**Figure 2**].** Peripherally released inflammatory signals derived from gut microbiota can prime microglia, thus stimulating iP upregulation in the CNS without the presence of amyloid plaques or tau tangles. The priming effect occurs via two different but possibly complementary routes (Lei et al. 2025). Firstly, the condition known as dysbiosis, disruption of gut microbiota balance, which is characteristic of both AD patients and animal models, is associated with increased gut permeability leaky gut and translocation of LPS and flagellin, products of gut microbiota, into the portal circulation (Lei et al. 2025). LPS and flagellin are recognized by toll-like receptors (TLR4 and TLR5) expressed not only by circulating monocytes but also by BBB endothelial cells (Acioglu and Elkabes 2025). As a result, an activation cascade occurs, resulting in cytokine production, such as IL-1β, TNF-α, and especially IFN-γ, which is known to be the strongest transcriptional inducer of iP subunit genes through the JAK-STAT1 pathway (Barnabei et al. 2021). Following this process, these cytokines can induce the expression of PSMB8, PSMB9, and PSMB10 proteins within microglia and perivascular macrophages and lower the threshold for Aβ/tau-induced neuroinflammation (Guo et al. 2022). Additionally, short-chain fatty acids (SCFAs), including butyrate, propionate, and acetate, produced by commensal bacteria via fermentation of dietary fibers, normally help in maintaining homeostasis of microglia by suppressing histone deacetylases (HDACs) and the NF-κB pathway (Ney et al. 2023). With respect to dysbiosis occurring in AD patients, the bacteria that produce SCFAs, such as certain Firmicutes, are reduced, thereby removing the suppressive mechanism on microglia and allowing microglial NF-κB to act synergistically with IFN-γ signaling to increase expression of iPs (Lei et al. 2025). This theory has been strongly supported in the preclinical model of germ-free 5xFAD mice with no microbiome and a decreased microglial expression of PSMB8 (60% decrease as compared to conventional 5xFAD mice), decreased tauopathy, and increased synapse density (Guilherme et al. 2021).

Surprisingly, colonization of these gnotobiotic mice with fecal samples from AD patients but not from control individuals reconstitutes iP expression in microglia and promotes faster tau propagation, providing clear evidence that there is indeed an effect on peripheral-to-CNS communication pathways (Ren et al. 2025). From a treatment point of view, the results presented show that peripheral administration of an orally bioavailable LMP7 inhibitor, one that cannot penetrate the CNS, may have beneficial effects on the disease by lowering inflammatory signals generated in the periphery and influencing CNS iP activity (Ren et al. 2025). Another way would be to target the microbiota through probiotics that generate SCFAs and even through dietary fiber, thereby lowering the amount of small molecule inhibitors needed to produce an effect and preventing any possible peripheral side effects (Markowiak-Kopeć and Śliżewska 2020).

## Therapeutic strategies

### Selective LMP7 inhibitors: from preclinical proof-of-concept to clinical candidates

One of the most advanced pharmacological approaches for regulating the immunoproteasome in AD includes selective inhibition of the β5i/LMP7 proteasome subunit. While first generation proteasome inhibitors like bortezomib inhibit both constitutive and immunoproteasomes without specificity causing unacceptable side effects in the brain and periphery, second generation compounds like ONX-0914 (PR-957) show over 100-fold preference for the inhibition of LMP7 subunit vs. constitutive β5 subunit inhibition (von Brzezinski et al. 2017). Chronic oral administration of ONX-0914 to 5XFAD mice, causing 40–60% LMP7 inhibition in the CNS, resulted in a 60–70% reduction in microglial senescence markers (p16^INK4a and SA-β-gal) and restored NRF2 protein levels, and decreased NLRP3 inflammasome activation (Shin et al. 2024). Indeed, the same regimen caused a significant reduction of exosomal tau forms in interstitial fluids by around 50% and also reduced trans-synaptic transmission of tau seeds from the injected hippocampus to the other hemisphere (Shin et al. 2024).

Recently, new-generation brain-permeable LMP7 inhibitors such as KZR-616 (now under clinical development as an immunosuppressive drug) and M3258 were found to be more potent in crossing the BBB with low IC50 values in the nanomolar concentration range (Park et al. 2024). A major finding from dose-finding experiments conducted on Tau-P301S transgenic mice is that total LMP7 blockade surprisingly accelerates tau aggregation by increasing cleavage specificity to produce stable 35-kDa seed-competent tau fragments, while partial LMP7 blockage maximizes both tau clearance and NRF2-dependent antioxidant effects (Takeuchi et al. 2011; Zhang et al. 2014).

### Dual LMP2/LMP7 modulation: harnessing proteasome-autophagy crosstalk

A second promising treatment strategy is based on the observation that the selective suppression of LMP7 function will not solve the concomitant disruption of autophagy-lysosome pathway function in AD patients. This is because the degradation of TFEB, the transcription factor regulating the biosynthesis of autophagosomes and lysosomes, occurs via its interaction with the β1i/LMP2 protein (Du et al. 2026). Therefore, a logical strategy to pursue would be a dual combination therapy involving the partial blockade of LMP2, in addition to LMP7 inhibition (Du et al. 2026). Preliminary testing of such an approach using a selective inhibitor of LMP2, LU-002i, showed that partial inhibition of LMP2 resulted in a tripling of the concentration of TFEB in microglial nuclei, increased LC3-II lipidation and cathepsin expression, and a decrease of around 30% in insoluble p-Tau aggregates in mice carrying the P301S mutant Tau gene (Bhattarai et al. 2020). Mechanistically, the dual inhibitor therapy works through the prevention of the degradation of p62/SQSTM1 by the LMP7 complex, which functions as an autophagy receptor connecting the ubiquinated tau oligomers to the LC3-positive autophagic vesicles (Bhattarai et al. 2020). Importantly, in this mechanism, the full inhibitory action against each individual subunit does not need to be achieved; instead, a certain level of basal activity of the proteasome is retained, while two different clearance pathways become active (Bhattarai et al. 2020). Medicinally, there are current ongoing efforts aimed at designing bifunctional inhibitors capable of binding to both LMP2 and LMP7 in different ratios, as well as developing fixed-dose combinations of subunit-selective inhibitors (Du et al. 2021). The major unknown point is whether this drug regimen could work effectively under intermittent dosing conditions such as two weeks on therapy and two weeks off therapy to avoid chronic alterations in immune system functioning and possible virus reactivations (Du et al. 2021).

### Peripheral and microbiome-based strategies to prime CNS immunoproteasome tuning

A completely different approach to treatment lies in regulating immunoproteasome function indirectly using the gut-brain axis, which does not require any drugs capable of CNS penetration and their neurotoxicity potential (Caetano-Silva et al. 2023). In patients with AD, gut dysbiosis leads to the depletion of the population of SCFA-producing bacteria (Firmicutes, Bacteroidetes), resulting in low concentrations of butyrate and propionate in serum and CSF. SCFAs inhibit microglia NF-κB pathway signaling and lower the expression of PSMB8 via histone deacetylases inhibition (Caetano-Silva et al. 2023). In 5XFAD mice, normalization of SCFA concentration in serum achieved by oral administration of sodium butyrate (200 mg/kg/day) lowered microglial LMP7 expression and inhibited p-Tau pathology (Jiang et al. 2021). Specifically, 12-week treatment of APP/PS1 mice with probiotics composed of bacteria strains capable of producing SCFAs (such as Clostridium butyricum, Lactobacillus plantarum) resulted in the significant lowering of LPS levels in plasma, decreased IFN-γ in CNS, and normalized PSMB8 expression in microglia associated with plaques (Hu et al. 2024).

Importantly, fecal microbiota transfer from young healthy donors to Tau-P301S mice was found to revert the microglia phenotype back to young cells and reduce the activity of tau-seeding in vitro (Loh et al. 2024). Therefore, peripheral LMP7 inhibitors unable to penetrate the BBB appear to be promising for development. This peripheral-to-central priming mechanism suggests that combination regimens pairing low-dose oral LMP7 inhibitors with SCFA-producing probiotics could achieve therapeutic CNS effects at one-third the drug exposure required for direct brain targeting, substantially lowering the risk of central adverse effects (Qian et al. 2022; Ravi et al. 2025).

## Limitations and future perspectives

Although convincing data support the use of the iP as a potential therapeutic target in AD, many caveats need to be addressed before pursuing this path further. First, most of the research has used transgenic animal models, including amyloid-overexpressing models such as 5xFAD or APP/PS1, as well as tauopathy models like Tau-P301S and PS19, which fail to completely capture the complex, multifaceted human disease that develops over decades and coexists with other diseases of aging. Aging itself induces the expression of iPs independent of any underlying AD pathology. It thus seems likely that the success of the therapy will vary greatly when tested on aged subjects rather than younger model organisms. Second, although the idea of tuning rather than inhibiting appears reasonable, the therapeutic window (40–60% β5i inhibition) is yet to be determined in humans and could turn out to be much smaller or nonexistent. Third, the long-term safety of chronic partial iP inhibition, potentially spanning years in a prodromal AD population, has not been evaluated beyond 12–16 weeks in animal studies. Risks include reactivation of latent neurotropic viruses (e.g., herpes simplex virus, varicella-zoster virus), impaired immune surveillance against CNS malignancies, and unexpected consequences for synaptic plasticity given emerging roles for proteasomal activity in long-term potentiation and memory consolidation. Fourth, the field lacks validated pharmacodynamic biomarkers that can be sampled repeatedly in humans to confirm that CNS β5i activity remains within the tuning window; plasma EV LMP7 activity is promising but requires extensive qualification.

In the future, some key areas of focus will arise. First, efficacy studies using physiologically relevant humanized aging mouse models (such as wild-type mice aged 18–24 months followed by tau seeding challenge) are necessary. Second, phase 1b trials in humans need adaptive dose-escalation designs including CSF sampling to determine the human PK-PD profile and tolerance to stepwise increases in β5i inhibition. Third, efforts in medicinal chemistry need to focus on non-covalent, reversible inhibitors with better brain uptake properties and shorter half-lives that allow rapid withdrawal in case of toxicity concerns. Fourth, the approach of intermittent dosing (for example, two weeks on, two weeks off) needs to be studied to avoid prolonged immunosuppression while providing therapeutic benefits. Fifth, taking into account the heterogeneity of AD, biomarkers (CSF iP activity, microglial senescence markers, p-Tau217) need to be implemented to select trials enriched with individuals having high baseline iP activity, as individuals with normal iP activity are not likely to benefit from iP modulation. The success in addressing these limitations will define whether the iP will become another validated target for AD or just another promising approach never to be exploited clinically.

## Conclusion

The discovery of the iP provides an unexplored nexus at the crossroads of tau proteinopathy, microglial senescence, and inflammation in AD, representing a novel strategy that is entirely separate from targeting amyloid or anti-inflammatories. The key message in this review is that the iP is not just a mediator of disease, which must be inhibited, or a guardian of health, which must be promoted, but rather a tightly controlled rheostat, which needs to be fine-tuned in the appropriate 40–60% range of selective inhibition of β5i/LMP7 subunits. Tuning, not silencing of this complex provides multiple advantages since it decreases microglia senescence through NRF2 maintenance, clears tau hyperphosphorylation without formation of seed-competent peptides, and preserves periphery immunity, all at once through the activation of a single pharmacologic target. The availability of CNS-penetrant subunit-specific inhibitors, validated CSF and plasma EV levels of LMP7 as biomarkers, and understanding how iP affects autophagy have generated a translational roadmap from the lab to the clinic.

With imminent first-in-man testing of selective inhibitors of LMP7 in early-stage AD, the community must be aware of the inherent difficulties with long-term manipulation of CNS immunity in general. Indeed, should this approach prove efficacious, not only would it present another treatment strategy for AD but could also set a blueprint for treating other aging-related neurodegenerative disorders such as tauopathies and Parkinson’s disease, where proteotoxicity and low-grade neuroinflammation intersect. A cautious optimism exists here: the biology and chemistry seem promising, while the unmet medical need is high.

## Data Availability

No datasets were generated or analysed during the current study.

## Abbreviations

5XFAD: Transgenic mouse model expressing five familial Alzheimer's disease mutations
AD: Alzheimer's disease
ADNI: Alzheimer's disease neuroimaging initiative
ALDH1L1: Aldehyde dehydrogenase 1 family member L1
AMPK: AMP-activated protein kinase
APOE: Apolipoprotein E
APP/PS1: Amyloid precursor protein/Presenilin 1
ATF6: Activating transcription factor 6
ATG5/7: Autophagy related 5/7
AUC: Area under the curve
Aβ: Amyloid-beta
BBB: Blood-brain barrier
CA1: Cornu Ammonis 1 (hippocampus region)
CD11b: Cluster of differentiation 11b
CD36: Cluster of differentiation 36
CD8+: Cluster of differentiation 8 positive (T cells)
CDR-Sum of Boxes: Clinical Dementia Rating Sum of Boxes
CNS: Central nervous system
cP: Constitutive proteasome
CSF: Cerebrospinal fluid
CST7: Cystatin F
CX3CR1: CX3C chemokine receptor 1
CXCL10: C-X-C motif chemokine ligand 10
DAM: Disease-associated microglia
DOI: Digital Object Identifier
ER: Endoplasmic reticulum
EVs: Extracellular vesicles
GFAP: Glial fibrillary acidic protein
HDACs: Histone deacetylases
HLA-A B C: Human leukocyte antigens A B and C
HO-1: Heme oxygenase-1
IC50: Half-maximal inhibitory concentration
IFIT1/2/3: Interferon-induced protein with tetratricopeptide repeats 1/2/3
IFN-gamma: Interferon-gamma
IL-1β: Interleukin-1 beta
IL-6: Interleukin-6
iP: Immunoproteasome
IRE1-α: Inositol-requiring enzyme 1 alpha
IRM: Interferon-response microglia
ISG15: Interferon-stimulated gene 15
JAK-STAT1: Janus kinase-signal transducer and activator of transcription 1
Keap1: Kelch-like ECH-associated protein 1
LC3: Microtubule-associated protein 1 light chain 3
LMP2: Low molecular mass polypeptide 2
LMP7: Low molecular mass polypeptide 7
LPL: Lipoprotein lipase
LPS: Lipopolysaccharide
MAP kinase: Mitogen-activated protein kinase
MCI: Mild cognitive impairment
MECL-1: Multicatalytic endopeptidase complex-like 1
MHC Class I: Major histocompatibility complex class I
miRNAs: MicroRNAs
mTOR: Mammalian target of rapamycin
mTORC1: Mammalian target of rapamycin complex 1
MX1: Myxovirus resistance protein 1
NAD(P)H: Nicotinamide adenine dinucleotide phosphate
NF-ҡB: Nuclear factor kappa-light-chain-enhancer of activated B cells
NLRP3: Nucleotide-binding oligomerization domain leucine-rich repeat and pyrin domain-containing 3
NQO1: NAD(P)H quinone dehydrogenase 1
NRF2: Nuclear factor erythroid 2-related factor 2
P2RY12: Purinergic receptor P2Y12
p-Tau: Phosphorylated tau
p16^INK4a: Protein 16 (inhibitor of kinase 4a)
p21^CIP1/WAF1: Protein 21 (cyclin-dependent kinase inhibitor 1/wild-type activating factor 1)
p62/SQSTM1: Sequestosome 1
PERK: Protein kinase RNA-like endoplasmic reticulum kinase
PK/PD: Pharmacokinetics/Pharmacodynamics
PROTACs: Proteolysis-targeting chimeras
PSMB8/9/10: Proteasome 20S subunit beta 8 9 and 10
ROS: Reactive oxygen species
SA-β-gal: Senescence-associated beta-galactosidase
SASP: Senescence-associated secretory phenotype
SCFAs: Short-chain fatty acids
STAT1: Signal transducer and activator of transcription 1
TFEB: Transcription factor EB
TLR4/5/7/8: Toll-like receptors 4 5 7 and 8
TMEM119: Transmembrane protein 119
TNF-α: Tumor necrosis factor-alpha
TREM2: Triggering receptor expressed on myeloid cells 2
UPR: Unfolded protein response
UPS: Ubiquitin-proteasome pathway/system
USP14: Ubiquitin-specific protease 14
